# Editorial: Short-Term Versus Long-Term Challenges in Functional Biomaterials Interfacing Living Systems: Two Sides of the Coin

**DOI:** 10.3389/fbioe.2021.723451

**Published:** 2021-08-04

**Authors:** Valentina Castagnola, Elisa Castagnola, Christian Bergaud, Davide Ricci

**Affiliations:** ^1^Center for Synaptic Neuroscience and Technology, Istituto Italiano di Tecnologia, Genoa, Italy; ^2^IRCCS Ospedale Policlinico San Martino, Genoa, Italy; ^3^Department of Bioengineering, University of Pittsburgh, Pittsburgh, PA, United States; ^4^MEMS Group, Laboratoire d'analyse et d'architecture des systèmes (LAAS-CNRS), University of Toulouse, Toulouse, France; ^5^DITEN, University of Genoa, Genoa, Italy

**Keywords:** functional biomaterial, nanomedicine, nanoparticle, neural interface devices, drug delivery & targeting

Functional biomaterials (FBMs) have been increasingly adopted as a key element in all sorts of biomedical devices for innovative diagnostic and therapeutic solutions, as they can be tailored for specific applications while being highly tolerated by living systems. Thanks to their versatility, FBMs have been used as vehicles for targeted pharmaceutical delivery to address cancer and tissue/bone degenerations, engineered as scaffolds for musculoskeletal, cardiovascular, and nerve regeneration, or employed as electrode coatings to enhance sensing/modulation of specific signals and stability, as well as biodegradable encapsulations/substrates for the attenuation of host tissue responses.

Tailoring biomaterial properties for such a plethora of applications is a challenge that requires a merging of knowledge across chemistry, pharmacy, biology, physics, and engineering.

Clinical applications often involve opposing requirements in terms of FBMs' lifetimes. In some cases, the FBMs should be stable and efficiently interact with the biological environment for many years after implantation, while in others they may need to be safely biodegraded or gradually bio-absorbed once their restorative or delivery functions have been accomplished. Sometimes a combination of the two approaches is desirable.

In this special issue we focus on a number of key aspects that allow to better understand interactions between biomaterials and living cells, improving our ability to tailor FBM properties for specific applications. The following topics are covered:

- effects of biomolecular corona on the nanomaterials' surface
- optimization of 3D *in vitro* models of human tissues
- strategies for long-term implants of neural devices for recording, detection, and drug delivery
- 1-D and 2-D nanomaterial interactions with living systems and the impact of the fabrication methods.

## Effects of Biomolecular Corona on the Nanomaterials' Surface

Nanoparticles represent an extraordinary tool for targeted therapeutics and diagnostics. Still, despite recent advances, only a few nanoformulations underwent successful clinical translation.

It is well-known that the discrepancy between *in vitro* and *in vivo* results originated from the formation of the biomolecular corona on the nanomaterials' surface, which defines a new biological identity masking the originally engineered surface chemistry. Therefore, the understanding of the mechanisms occurring at the nanomaterials-cell interface, mediated by the biomolecular corona, is a priority for a fast translation of functional nanomaterials into clinical applications.

The review article “Disentangling biomolecular corona interactions with cell receptors and implications for targeting of nanomedicines” of Aliyandi et al., gives an overview of our knowledge to date on the mechanisms of biocorona interaction with the cell machinery with the aim of exploiting it to develop novel targeting strategies.

**Figure 1:**
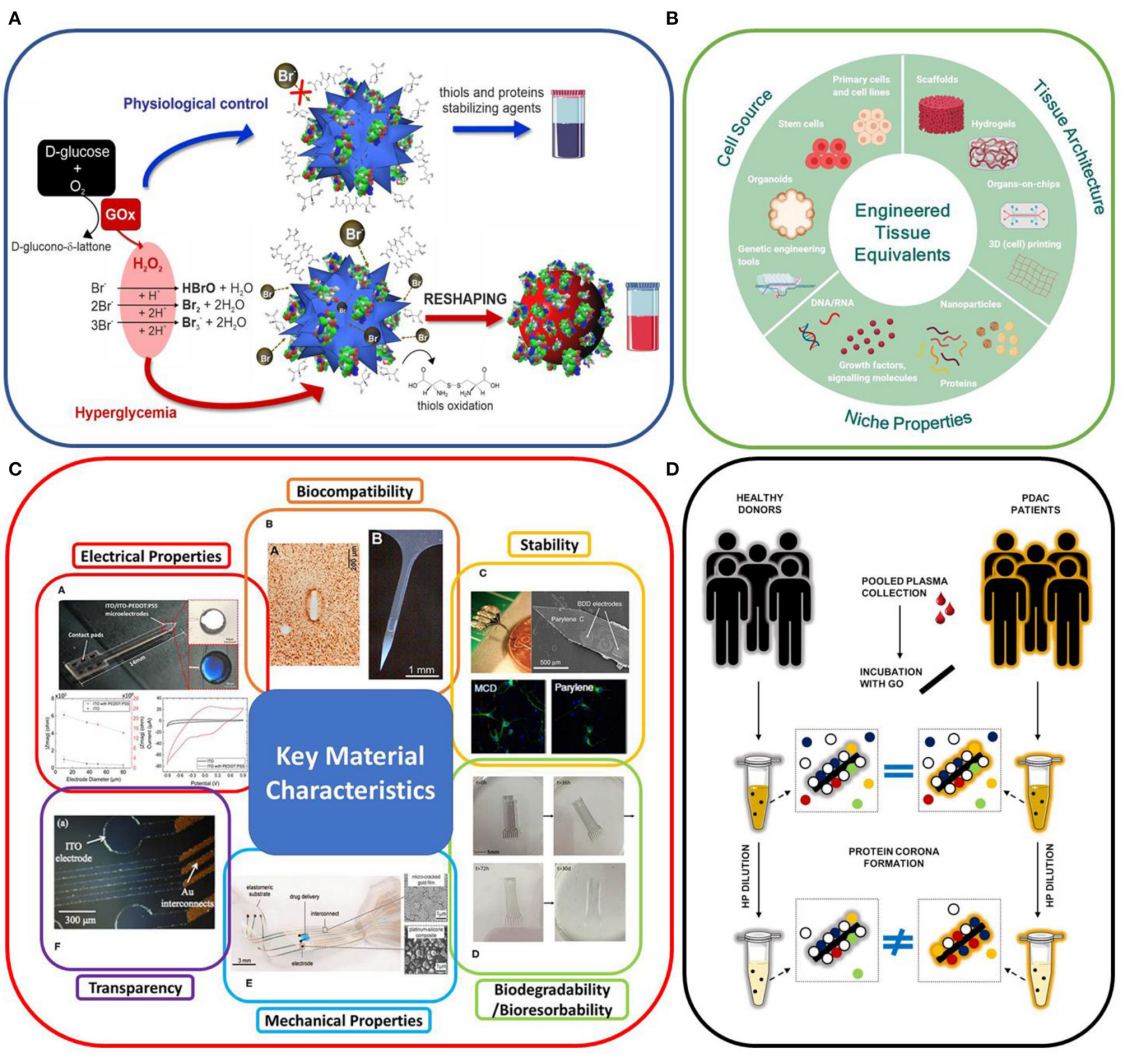
**(A)***in vitro* colorimetric assay using highly responsive multibranched gold nanoparticles (MGNPs) suspended in saliva: schematics of the mechanisms involved. From Donati et al. **(B)** Engineering human tissue equivalents *in vitro*; from Moysidou et al. **(C)** Key material characteristics of neural recording implants. From Yang et al. **(D)** Cartoon describing the present understanding of the role of protein concentration on personalization of protein corona from Di Santo et al.

While the biomolecular corona represents a challenge in nanomedicine, it can also be turned into a weapon in the field of diagnostics. In the article “Colorimetric nanoplasmonics to spot hyperglycemia from saliva” of Donati et al. a non-invasive colorimetric (naked-eye) sensing platform to identify hyperglycemia from saliva is reported, exploiting the plasmonic properties of gold nanostructures. Here the protein corona forming from biomolecules present in saliva seems to play a role in improving the performances and promoting the reshaping process responsible for the colorimetric change (Figure 1A).

## Optimization of 3D *in vitro* Models of Human Tissues

Parallel efforts to reduce the *in vitro/in vivo* mismatch must be made on the biological models. The review article “Advances in Engineering Human Tissue Models” of Moysidou et al. (Figure 1B) covers the recent advances in 3D *in vitro* models of human tissues, highlighting the fact that this technology has the potential to fill the gap between *in vivo* studies and human physiological and diseased models, a necessary step for clinical translation. In the article “Hydrogels for 3D neural tissue models: Understanding cell-material interactions at a molecular level” of Vallejo-Giraldo et al. a poly(vinyl alcohol) biosynthetic hydrogel functionalized with gelatin and sericin was used to promote the development of complex neural networks. Here the role of the astrocytic support on the neural outgrowth was analyzed at the molecular level, giving important indications for future hydrogels designs.

## Strategies for Long-Term Implants of Neural Devices for Recording, Detection, and Drug Delivery

It is well-known that the main challenge faced by implantable neural devices is their long-term functionality and biocompatibility. The implant instability is mainly attributed to the foreign body response, neural degeneration, poor long-term material functionality and biocompatibility, mechanical mismatch between the brain tissue and the implant (Fattahi et al., 2014; Wellman et al., 2018). The review article “Foreign body reaction to implanted biomaterials and its impact in nerve neuroprosthetics” of Carnicer-Lombarte et al. provides an extensive summary of the current nerve neuroprosthetic technologies and discusses how their long-term functional stability is limited by the foreign body reaction (FBR). Additionally, it provides an overview of different strategies, such as material properties, pharmacological therapies, or the use of biodegradable materials, that may be exploited to minimize FBR and improve the long-term stability of neuroprosthetic nerve interfaces.

The choice of the material for device fabrication and the surgical delivery procedure become a critical factor in minimizing the damage to the tissue and providing a reliable and accurate functionality of a neural interface. Different intrinsic material properties (strength, flexibility, conductivity, and chemical inertness) and design parameters (geometry, size, packaging) must be considered. The review article “A Review: Electrode and Packaging Materials for Neurophysiology Recording Implants” of Yang et al. summarizes the state of the art of neural tissue implants for neurophysiological recording *in vivo* (Figure 1C). Particular attention has been dedicated to reviewing the different strategies adopted to tune the structural, functional, and dimensional properties of the different components of the implanted devices with the aim to match the mechanical, chemical, and electrical properties of the nervous system, improving the device–tissue interaction and longevity.

The research article “Explant analysis of recording and stimulating Utah electrode arrays implanted in human cortex for brain-computer-interfaces,” focuses on the evaluation of the long-term stability of Utah electrode arrays implanted in the human brain for bi-directional brain-computer interface application. Specifically, the authors examined six explanted arrays from two human participants and assessed the degrees of material degradation and fibrous encapsulation. Changes in the extent of material degradation and encapsulation were observed based on the length of implantation, but these changes did not correlate with recording performance prior to explantation.

The research article “Biocompatibility of a Conjugated Polymer Retinal Prosthesis in the Domestic Pig” of Maya-Vetencourt et al., reports about their progress toward human translation of biocompatible conjugated polymer retinal prosthesis, previously tested in rodents. They detailed the fabrication and *in vivo* testing of multi-layer fully organic prosthesis implanted subretinally in the eye of domestic pigs. Their findings highlight the biocompatibility of this new generation of retinal prosthesis and their potential suitability for subretinal implantation in patients suffering from degenerative blindness.

When a device is interfaced with deep brain structures for electrochemical neurotransmitter sensing, there are additional aspects to be considered, such as achieving stable sensitivity *in vivo*. Thus, the microelectrodes need to be electrochemically stable and resistant to chemical and biological fouling, other than presenting good adsorption properties and fast electron transfer kinetics. The research article “Real-Time Fast Scan Cyclic Voltammetry Detection and Quantification of Exogenously Administered Melatonin in Mice Brain” of Castagnola et al., shows a successful real-time drift-free detection of exogenous administered MT in mouse brain using fast-scan cyclic voltammetry (FSCV) at carbon-fiber microelectrodes (CFEs). A prolonged CFE pre-conditioning was demonstrated to stabilize the FSCV background currents, both *in vitro* and *in vivo*, and to provide consistent CFE sensitivity over time, even in the presence of a high MT concentration. In alternative to electrochemical methods, microdialysis is a powerful tool for the measurement and understanding of the neurochemistry of the brain. The review article “Dexamethasone-Enhanced Microdialysis and Penetration Injury” of Jaquins-Gerstl and Michael summarizes the microdialysis principle, they discuss the immune response resulting from microdialysis probe implantation, and the use of dexamethasone (DEX) -enhanced microdialysis to stabilize the surrounding tissue allowing for better detection of chemical species, attenuated microglia and reduce gliosis.

Finally, the article “Microfluidic Multielectrode Arrays for Spatially Localized Drug Delivery and Electrical Recordings of Primary Neuronal Cultures” of Bruno et al. calized drug delivery and cell signaling recording of spontaneous and chemically stimulated activity in primary neuronal networks. This platform shows the capability to examine the effect of biochemical agents on the desired portion of cell culture.

## 1-D and 2-D Nanomaterial Interactions With Living Systems and the Impact of the Fabrication Methods

In recent years 1-D and 2-D nanomaterials have been increasingly proposed as functional biomaterials offering an extremely wide spectrum of potential applications that rely not only on contrasting properties such as short or long-term lifetime, but even on opposite biological effects such as cytotoxicity vs. promotion of cell proliferation and differentiation. For this reason, they offer a very interesting case-study for investigating mechanisms of interaction with living systems.

The review article “Interactions Between 2D Materials and Living Matter: A Review on Graphene and Hexagonal Boron Nitride Coatings” of Santos et al. addresses the fundamental question on why two-dimensional material coatings exhibit complex and controversial interactions with biological matter, having shown in different contexts to induce bacterial cell death and contribute to mammalian cell growth and proliferation *in vitro* and tissue differentiation *in vivo*. After considering morphological, chemical, and electronic properties of materials described in state-of the-art literature, a picture of their correlation with functionality emerges, usefully guiding future applications.

More specifically, the implications of a 2-D nanomaterial size (graphene oxide) on anti-cancer applications is addressed in the review article “Methods to Scale Down Graphene Oxide Size and Size Implication in Anti-cancer Applications” of Tufano et al. It presents a thorough description of the main methods to reduce and homogenize in nanometric scale the lateral dimensions of graphene oxide, either during production or *via* post-processing, together with a discussion of the implication of the small size in cancer treatment by exploiting GO nanocarriers as an effective theragnostic tool. On the same subject of graphene oxide, the original research paper “Personalized Graphene Oxide-Protein Corona in the Human Plasma of Pancreatic Cancer Patients” of Di Santo et al., provides a proof of concept that exposing nanomaterials to plasma samples under “optimal dilution conditions” could be a good strategy to amplify personalization of protein corona and, in turn, to exploit it to distinguish between different classes of donors (e.g., cancer vs. non-cancer patients) (Figure 1D).

One of the greatest challenges for exploiting nanomaterials in living beings is the development of appropriate pathways for their biofunctionalization. The original research work presented in “A Plant Bioreactor for the Synthesis of Carbon Nanotube Bionic Nanocomposites” of Magnabosco et al., by incorporating functionalized single-walled carbon nanotubes within the roots of living plants producing a bionic composite material, poses the basis for *in vivo* synthesis of new materials taking advantage of the living organism as a reactor.

At last, the key issue of ensuring the ability to directly monitor and evaluate the interactions of the novel nanoparticle (NP) based functional materials with living systems is addressed in the review “*in vitro* Label Free Raman Microspectroscopic Analysis to Monitor the Uptake, Fate and Impacts of Nanoparticle Based Materials” of Byrne et al., were it is demonstrated that the—well-known to material scientists—Raman Microspectroscopy technique, is capable of confirming the intracellular localization of NPs in cells, monitoring the NP trafficking in subcellular vesicles and, monitoring NP degradation catabolization, identifying and tracking cellular response pathways, all in a single, label-free, measurement protocol.

## Author Contributions

All authors listed have made a substantial, direct and intellectual contribution to the work, and approved it for publication.

## Conflict of Interest

The authors declare that the research was conducted in the absence of any commercial or financial relationships that could be construed as a potential conflict of interest.

## Publisher's Note

All claims expressed in this article are solely those of the authors and do not necessarily represent those of their affiliated organizations, or those of the publisher, the editors and the reviewers. Any product that may be evaluated in this article, or claim that may be made by its manufacturer, is not guaranteed or endorsed by the publisher.
